# Ferroptosis—A Novel Mechanism With Multifaceted Actions on Stroke

**DOI:** 10.3389/fneur.2022.881809

**Published:** 2022-04-11

**Authors:** Xiao-Ling Fang, Shao-Yun Ding, Xiao-Zheng Du, Jin-Hai Wang, Xing-Lan Li

**Affiliations:** ^1^College of Acupuncture and Massage, Gansu University of Traditional Chinese Medicine, Lanzhou, China; ^2^Department of Traditional Chinese Medicine, The Second Hospital of Lanzhou University, Lanzhou, China

**Keywords:** stroke, ferroptosis, iron overload, amino acid metabolism, lipid peroxidation, inflammation, autophagy, apoptosis

## Abstract

As a neurological disease with high morbidity, disability, and mortality, the pathological mechanism underlying stroke involves complex processes such as neuroinflammation, oxidative stress, apoptosis, autophagy, and excitotoxicity; but the related research on these molecular mechanisms has not been effectively applied in clinical practice. As a form of iron-dependent regulated cell death, ferroptosis was first discovered in the pathological process of cancer, but recent studies have shown that ferroptosis is closely related to the onset and development of stroke. Therefore, a deeper understanding of the relationship between ferroptosis and stroke may lead to more effective treatment strategies. Herein, we reviewed the mechanism(s) underlying the onset of ferroptosis in stroke, the potential role of ferroptosis in stroke, and the crosstalk between ferroptosis and other pathological mechanisms. This will further deepen our understanding of ferroptosis and provide new approaches to the treatment of stroke.

## Introduction

Stroke can be divided into two categories: ischemic stroke and hemorrhagic stroke. Ischemic stroke is caused by interruption of the cerebral blood supply, and hemorrhagic stroke results from cerebrovascular rupture or an abnormal vascular structure. Stroke is the second leading cause of death worldwide, with both high incidence and disability rates, and is extremely debilitating to human health and life, creating a heavy burden for a patient's family and society as a whole (1). With both younger individuals beset with the disease and the burgeoning of the aging population, the number of people who fall ill and die of stroke has increased significantly each year. If effective measures are not taken in a timely manner, the burden of stroke worldwide will continue to escalate, particularly in low-income countries. Therefore, it is incumbent on us to develop novel methods for the prevention and treatment of stroke. Emergent treatment using western medications and neurologic approaches has reduced the mortality rate attributable to stroke, but these modalities are often unable to prevent or reverse neuronal injury. As a result, surviving patients are often afflicted with sequelae such as hemiplegia, aphasia, affective disorder, and cognitive impairment, all of which seriously affect the quality of a patient's life (2–4). Therefore, the development of effective strategies for targeted neuroprotection would achieve an unprecedented breakthrough in this field.

The pathological mechanism of stroke involves complex processes such as neuroinflammation, oxidative stress, apoptosis, autophagy, and excitotoxicity; but the study of these molecular mechanisms has not yet been effectively applied in the clinic. Recent analyses, however, have shown that ferroptosis is closely related to the onset and development of stroke.

As a form of iron-dependent, regulated cell death, ferroptosis differs from other types of programmed cell death such as apoptosis and necrosis. Ferroptosis exhibits unique morphological and biochemical changes in which mitochondria are shrunken and the nucleus remains intact, thus distinguishing it from other forms of cell death and allowing its use as a specific marker (5). Moreover, this mode of cell death can be triggered by excitotoxic stimulation that is related to the up-regulation of intracellular reactive oxygen species (ROS) levels and can be reversed by iron- chelating agents or gene inhibition that blocks a cell's iron uptake (6, 7). It has been shown that the occurrence of ferroptosis is related to the metabolic processes of iron, amino acid, and lipid peroxidation, and is involved in complex pathological linkages such as the dysfunction of cysteine-glutamate antiporter system (system Xc^−^) (8), inactivation of glutathione peroxidase 4 (GPX4) (9), increases in the expression of acyl-CoA synthetase long-chain family member 4 (ACSL4) and lysophosphatidylcholine acyltransferase-3 (LPCAT3) (10, 11), and the up-regulation of lipoxygenase (LOX) levels (12). The most basic biochemical characteristics are iron overload and a lethal accumulation of intracellular lipid peroxides and ROS (13). Investigators have confirmed that ferroptosis is involved in the onset and development of many diseases in the body, e.g., ferroptosis plays an important role in nervous system diseases (14–17). The close relationship between ferroptosis and stroke has become increasingly apparent from recent studies and has therefore attracted widespread attention. Accumulating experimental data show that ferroptosis may be an effective neuroprotective target that can be used in novel approaches to the clinical treatment of stroke (18, 19). Herein, we will therefore review the mechanism(s) underlying ferroptosis in stroke, present the potential role of ferroptosis in stroke, and evaluate the crosstalk between ferroptosis and other pathological mechanisms with respect to this disease. Such information will further deepen our understanding of ferroptosis and provide novel approaches to the treatment of stroke.

## Mechanisms Underlying Ferroptotic Actions in Stroke

**Figure 1:**
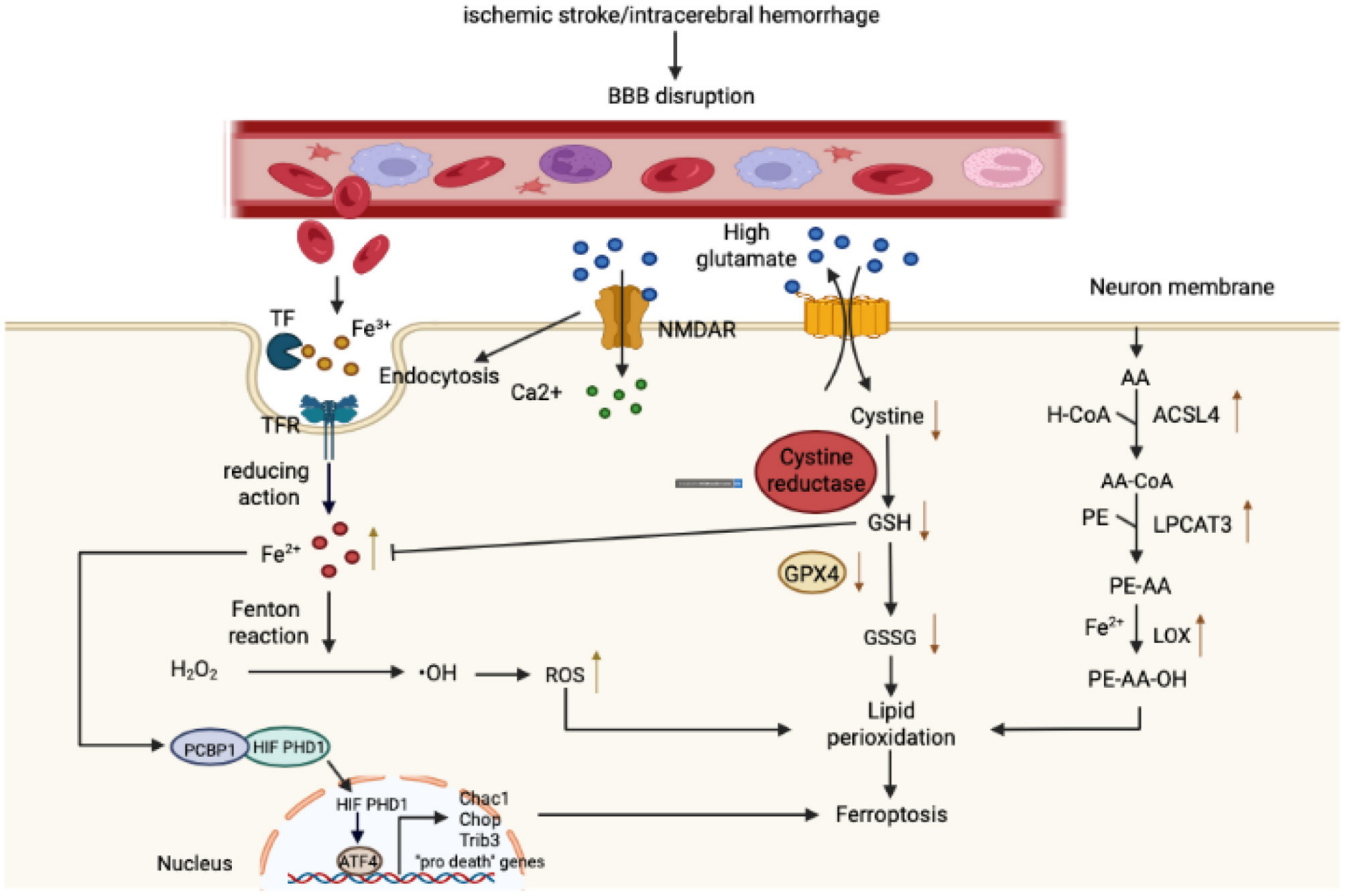
Mechanism governing ferroptosis in stroke. With respect to iron metabolism, after ischemic or hemorrhagic stroke, the permeability of the blood-brain barrier (BBB) increases, causing a variety of components rich in Fe^3+^ in the bloodstream to infiltrate into the brain parenchyma. Fe^3+^ binds closely to transferrin (T) to form iron-containing TF, which then binds to TFR1 on the surface of brain cell membranes, enters cells through pinocytosis, and forms an endosome. In the acidic environment of an inclusion body, Fe^3+^ is released from TF, catalyzed by ferrous reductase to Fe^2+^, and transported to the cytoplasm through the corresponding transporter. Fe^2+^ then initiates the Fenton reaction to form reactive oxygen species (ROS) and also affects the catalytic activity of lipoxygenase (LOX). In addition, the Fe^2+^ in the cytoplasm can be absorbed by PCBP1 and iron is loaded on the iron-free form of HIF-PDH1. Iron-containing HIF-PDH1 together with other stimuli drives the activity of pro-ferroptosis ATF4 gene expression. With regard to amino acid metabolism, a high concentration of extracellular glutamate leads to a dysfunction in system XC^−^, a deficiency in intracellular cystine, depletion of GSH, and diminution in GPX4 activity. In addition, glutamate binds to its receptor (NMDAR), and the activation of NMDAR then further exacerbates iron uptake. Regarding lipid metabolism, as the main component of phospholipid membranes, arachidonic acid (AA) is esterified with phosphatidylethanolamine (PE) under the action of ACSL4 and LPCAT3 to synthesize PE-AA and is then catalyzed by LOX to PE-AA-OH. As shown in the figure, these three components together lead to lipid peroxidation and ferroptosis.

As mentioned previously, the metabolism of iron, amino acids, and lipids constitutes the three major pathways leading to ferroptosis. We will therefore analyze and summarize the regulatory mechanism underlying ferroptosis in stroke from these three aspects (Figure 1).

### Iron Metabolism in Stroke

#### Iron Overload and Stroke

Iron is a metal with redox activity that participates in the formation of ROS and the diffusion of lipid peroxidation products, and elevated iron levels increase the susceptibility of cells to iron-dependent cell death (20). Intracellular iron overload is thus the key to ferroptosis (21). In hemorrhagic stroke and the late stage of cerebral ischemia, ferroptosis occurs when blood rich in free iron and ferritin infiltrates into the brain parenchyma through a damaged blood-brain barrier (BBB).

Before the concept of ferroptosis was first realized, investigators demonstrated that iron overload and up-regulated expression of iron-handling proteins in the brain after intracerebral hemorrhage mediated toxic reactions that led to brain damage (22, 23). The data showed that iron overload could lead to mitochondrial breakage of hippocampal neurons (24), which significantly aggravated the early brain injury caused by intracerebral hemorrhage, and that the outcome could be reversed by treatment with iron-chelating agents (25, 26). There are two possible causes of iron overload in intracerebral hemorrhage. One cause is the inflammatory activation of the Toll-like receptor 4/myeloid differentiation factor (TLR4/MyD88)-signaling pathway after intracerebral hemorrhage. This promotes the expression of interleukin (IL-6), which activates the JAK/STAT3 pathway and thereby increases the expression of hepcidin, which in turn prevents iron from flowing out of the brain tissue into the bloodstream and leads to iron overload in the brain (27, 28). The other cause is the heme and globin formed by erythrocyte lysis after intracerebral hemorrhage, after which heme is degraded into iron ion, bilirubin, and CO under the action of heme oxygenase-1 (HO-1). Studies have shown that HO-1 increases significantly and leads to iron overload after intracerebral hemorrhage and that HO-1 inhibitors can reduce the damage of cerebral hemorrhage in rats and pigs (16).

Brain iron disorders are also the key to neuronal death after ischemic stroke (29, 30). Ferroptosis caused by iron overload after cerebral ischemia exacerbates mitochondrial oxidative damage and enlarges the volume of cerebral infarctions (31, 32). Children afflicted with hypoxic-ischemic encephalopathy manifest iron deposits in the affected basal ganglia, thalamus, and periventricular white matter (33). In addition, brain iron accumulates during the process of normal aging, and aging is the first risk factor for stroke, with the two occurring concomitantly (14). It has been found that hepcidin inhibits the release of iron by binding to ferroportin1 (FPN1) on cell membranes and induces the internalization and degradation of the protein, and the increase in serum hepcidin and iron concentration in patients with ischemic stroke indicates that hepcidin plays an important role in ischemic iron overload of the cerebrum (34). Recent studies have shown that iron overload after ischemic stroke may be affected by the following two mechanisms. One is the up-regulation of IL-6 expression after cerebral ischemia—as IL-6 regulates the JAK/STAT3 pathway to increase hepcidin content, down-regulate FPN1, and inhibit iron output—resulting in iron overload (35). The second mechanism is an increase in the expression of hypoxia-inducible factor 1α (HIF-1α) in the focal brain tissue, which induces the up-regulation of transferrin receptor 1 (TFR1) and leads to the accumulation of iron. Previous studies have demonstrated that iron uptake in neurons was principally dependent on the TF-TFR1 pathway, suggesting that HIF-1 receptor-mediated TFR1 expression may comprise another cause of iron overload after cerebral ischemia (36, 37). Thus, iron overload after ischemic and hemorrhagic stroke exhibits a common mechanism, i.e., the up-regulation of IL-6 leading to the elevation of ferritin expression through the JAK/STAT3 pathway, resulting in the down-regulation of cellular FPN1 and the diminution of iron efflux from neurons. However, the mechanism(s) subserving the up-regulation of IL-6 during cerebral ischemia remains unclear.

The use of an iron-chelating agent can inhibit iron overload and reduce stroke injury. It was reported that intraperitoneal injection of deferoxamine (DFO) induced focal cerebral ischemic tolerance, reduced the size of cerebral infarction, improved neurological function, and protected cultured cortical neurons from oxygen-glucose deprivation (OGD) (38). However, intranasal injection (39) can increase the concentration of DFO in the brain quickly, and this exerts a greater protective effect on the brain. In addition, studies have revealed that low-dose, long-term administration of DFO or deferriamine (DFR) maintained a long-term neuroprotective state in mice (40). Another iron-chelating agent-−2,2'-bipyridyl—significantly reduced the volume of cerebral infarction and lessened ischemic damage in endothelial cells and neurons in rats with permanent middle cerebral artery occlusion (pMCAO) (41). In a model of intracerebral hemorrhage, DFR also inhibited the expression of ferritin after intracerebral hemorrhage and hemolysis, reduced iron uptake, alleviated iron-mediated neuronal injury, and improved the prognosis of intracerebral hemorrhage (42, 43). Therefore, iron overload comprises an important regulatory mechanism underlying cell death from iron overload after stroke.

#### Iron Metabolism-Related Proteins and Stroke

Iron metabolism iron metabolism mainly involves iron influx and iron outflow, and these two processes largely depend on iron metabolism-related proteins including transferrin (TF), TFR1, divalent metal transporter (DMT1), ferritin (FT) and FPN1, with the activity of these proteins depending largely on the concentration of iron (44). Iron-regulatory proteins (IRPs) are a type of protein that can bind to the iron-responsive elements (IREs) of target mRNAs and control their functions, thus regulating the input and output of iron to maintain iron homeostasis (45, 46). Studies have also shown that the expression of iron uptake and iron-storage proteins increases during iron overload; heme iron in the brain increases significantly within a few weeks after intracerebral hemorrhage, and the levels of TF, TFR, and FT in the brain are augmented within a few days of intracerebral hemorrhage (22). A recent report indicates that TF can quickly enter the bloodstream from the brain through the BBB (47), suggesting that TF and TFR may exert dual effects on iron overload, i.e., they may not only cause iron overload but may also promote iron clearance in the brain when iron overload occurs. There is also a significant positive correlation between serum FT and the amount of edema around a hematoma; the higher the serum FT levels, the poorer the prognosis for stroke patients, indicating that iron toxicity has a significant influence on hemorrhagic brain injury and edema formation (48, 49). In addition, TF is up-regulated after ischemic stroke and results in neuronal damage (50), which shows that the role portrayed by the up-regulation of TF and TFR after stroke is unclear; this phenomenon contradicts the aforementioned iron-homeostatic regulation and indicates the presence of another unknown regulatory mechanism.

### Amino Acid Metabolism in Stroke

#### System Xc^–^ and Stroke

The system Xc^−^ cysteine/glutamate antiporter is a heterodimer composed of solute carrier family 3 member 2 (the SLC3A2 regulatory subunit) and SLC7A11(the solute carrier 7A11, also known as xCT; a subunit that acts as an amino acid transporter) (5). System Xc^−^ regulates intracellular glutathione (GSH) to eliminate excess hydroxyl peroxides, and after stroke, glutamate (Glu) uptake decreases and its release increases, resulting in an elevation in extracellular Glu concentration and subsequent excitotoxicity (51); this action then blocks the reverse transport function of system Xc^−^ and causes ferroptosis (52). However, the expression of SLC7A11 is up-regulated in most animal models of nervous system diseases (53). Data show that HIF-1α up-regulates SLC7A11 and induces damage by binding to its promoter, while SLC7A11 gene knockout in mice inhibits brain injury induced by cerebral ischemia/reperfusion (CI/R) or oxygen-glucose deprivation/reoxygenation (OGD/R) *in vivo* and *in vitro* (54). The above experimental results seem to violate our logic. On the one hand, SLC7A11 is a part of antiporter in Xc- system, but what is puzzling is that it induces damage after up-regulation. We speculate that under normal conditions, the expression level and functional state of SLC3A2 and SLC7A11 are in balance to maintain the normal transport function of Xc^−^ system. When SLC7A11 is up-regulated, it breaks the balance between them, thus inducing damage. In addition, N-acetylcysteine (NAC) and ceftriaxone (CEF) interfered with MCAO rats to down-regulate the expression of SLC7A11 in neurons and astrocytes and induced cerebral ischemic tolerance (55). Therefore, system Xc^−^ mediates ferroptosis after ischemic stroke, but it is still uncertain whether it is involved in the regulation of neuronal ferroptosis after hemorrhagic stroke, and the role of system Xc^−^ in glutamate toxicity and ferroptosis after stroke remains arcane and requires further exploration.

#### GPX4, GSH and Stroke

Reduced GSH is an important co-factor in the scavenging of membrane lipid peroxides by glutathione peroxidase 4 (GPX4), thereby reducing oxidative stress (56). Under pathological conditions, intracellular GSH depletion and GPX4 inactivation lead to ferroptosis (13), and GSH and GPX4 are the key regulators of ferroptosis and are closely related to hemorrhagic and ischemic stroke. Researchers have found that the expression level of GPX4 and GSH was inhibited after stroke and that selenium promoted the expression of GPX4 by activating the transcription factors TFAP2c and Sp1; this effectively inhibited ferroptosis and Glu excitotoxicity, thus reducing hemorrhage or ischemic brain injury in mice (57). Carvanol attenuated neuronal damage in ischemic gerbils by increasing the expression of GPX4 (58); treatment with naotaifang extract (NTE) up-regulated the contents of GPX4 and GSH in the brain of MCAO rats, improving functional outcome (59); and kaempferol played a neuroprotective role by activating the Nrf2/SLC7A11/GPX4-signaling pathway, enhancing antioxidant capacity, and reversing OGD/R-induced ferroptosis (60). In the acute phase of intracerebral hemorrhage, the expression levels of GPX4 in neurons decreased significantly, while the enhancement of its gene expression significantly inhibited the onset of neuronal ferroptosis and improved the prognosis of intracerebral hemorrhage in rats. Interference with or knockout of the GPX4 gene conversely aggravated the brain injury (61). Dauricine also attenuated secondary brain injury after intracerebral hemorrhage by up-regulating the co-expression of GPX4 and glutathione reductase (GSR) to inhibit neuronal ferroptosis (62). In a subarachnoid hemorrhage (SAH) model *in vivo* and in vitro, overexpression of GPX4 significantly reduced lipid peroxidation in neurons, improved brain edema and neurological impairment after SAH, and showed critical protection of the brain (63).

As one of the key regulators of ferroptosis, GSH not only functions in an auxiliary capacity in the activity of GPX4 but is also the only remaining ligand of Fe^2+^ in cells that can block the Fenton reaction and reduce the production of highly toxic hydroxyl radicals so as to inhibit ferroptosis (64). Studies have shown that edaravone—a free radical scavenger approved clinically for the treatment of ischemic stroke and amyotrophic lateral sclerosis (ALS)—resists ferroptosis caused by various pathological factors, of which the therapeutic effect achieved by GSH deprivation on ferroptosis is the most pronounced (65). The above data showed that under the pathological conditions of stroke, GSH depletion and GPX4 inactivation led to ferroptosis, while increasing GSH synthesis and promoting GPX4 activity inhibited the onset of ferroptosis and reduced the injury caused by hemorrhage and ischemic stroke. However, current research is focused primarily on the effects of GPX4 and GSH on stroke, and a large gap persists in our understanding of the upstream regulatory mechanisms; this therefore requires further investigation.

### Lipid Peroxidation in Stroke

#### LOX and Stroke

LOX is a key enzyme that catalyzes lipid peroxidation and ferroptosis caused by polyunsaturated fatty acids (PUFAs) in phospholipid membranes (12), and has a variety of subtypes. We presently possess a relatively clear understanding of 12/15-LOX and 5-LOX. It was reported that 12/15-LOX was highly expressed in the pMCAO mouse model and that its inhibitor LOX Block-1 (LB-1) inhibited ferroptosis and significantly reduced infarct volume. Similarly, in an ischemia/thrombolysis model, LB-1 treatment not only decreased neuronal mortality but also attenuated the bleeding area (66). In a global cerebral-ischemia model, the 12/15-LOX in the vascular system and neurons in the cortex and hippocampus increased in a time-dependent manner, and gene knockout or the use of LB1 reduced brain injury and improved recovery (67). However, after intracerebral hemorrhage, the activity of LOX-5 increases, which induces the production of lipid peroxides and leads to ferroptosis (18). N-acetylcysteine (NAC) prevents heme-induced ferroptosis in neurons by neutralizing toxic lipids produced by nuclear LOX-5 (18). Therefore, LOX mediates ferroptosis after stroke, and the inhibition of LOX reduces ischemic and hemorrhagic brain injury. However, how lipoxygenase subtypes cooperate to participate in lipid peroxidation and induce ferroptosis after stroke requires further elucidation.

#### ACSL4 and Stroke

ACSL4 shapes the lipid composition of cells, affects the synthesis of lipid peroxides, and determines the sensitivity of cells to ferroptosis (11). ACSL4, which is widely expressed in the brain tissue, is a newly discovered and enigmatic ischemia-related protein. It has been used as a potential target regulated by miR-347 during cerebral ischemia, and its mechanism of action may be the overexpression of miR-347 after cerebral ischemia; this then indirectly regulates ACSL4 through transcription to mediate neuronal ferroptosis (68). Chen et al. (69) observed increased expression of ACSL4 protein in the MCAO mouse model, and rosiglitazone inhibited its activity and regulated ferroptosis, thus promoting the recovery of neurologic function after stroke. In addition, paeonol significantly inhibited ferroptosis in an extracorporeal cerebral-hemorrhage model and controlled the progression of cerebral hemorrhage by down-regulating the HOTAIR/UPF1/ACSL4 axis (70). The level of ACSL4 in the brain tissue of rats with SAH was significantly increased; however, the use of siRNA to inhibit the expression of ACSL4 alleviated inflammation, BBB damage, brain edema, and behavioral and cognitive impairment after SAH by promoting neuronal survival (71). Although ACSL4 is an important factor in ferroptosis after stroke, the research in this field remains limited. We, therefore, need to further explore its underlying mechanism(s) of action.

## Crosstalk Between Ferroptosis and Other Pathologic Processes in Stroke

**Figure 2:**
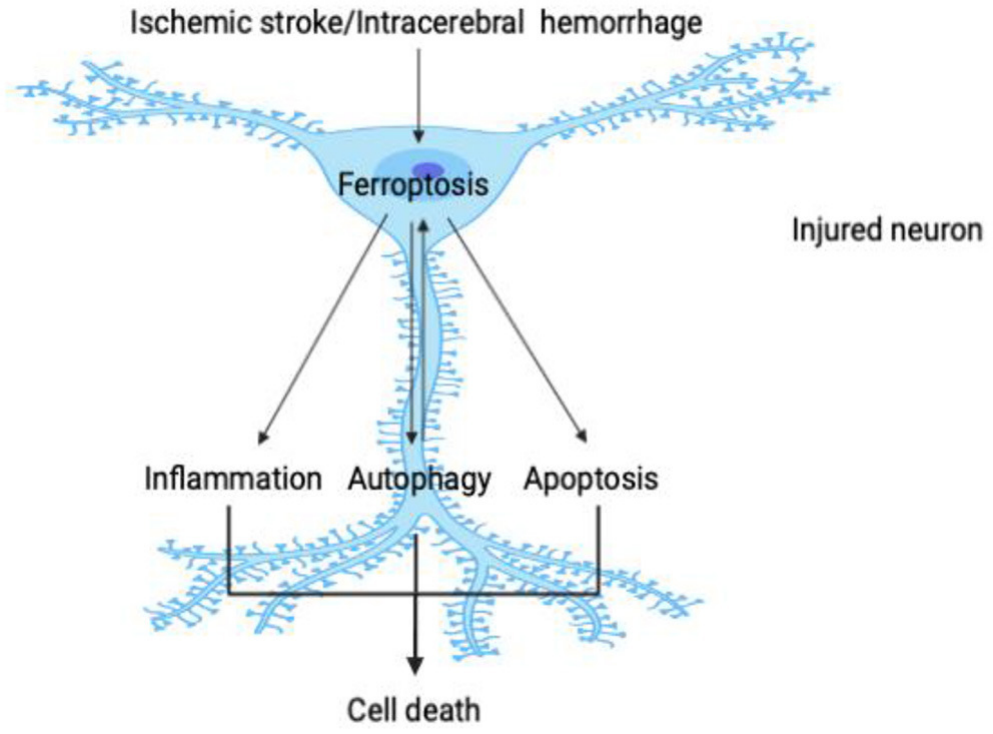
Crosstalk between ferroptosis and other pathological processes in stroke. As shown in the figure, the pathological stimulation caused by stroke causes ferroptosis in damaged neurons; and as one of the core events in cell injury, ferroptosis can then further promote neuroinflammation, autophagy, and apoptosis. Ferroptosis is triggered interactively with these pathological processes and participates in a close one-way or even two-way relationship so that, in the process of stroke, these key constituents initiate a variety of cascade reactions and jointly determine the survival of neurons.

Brain injury caused by stroke manifests multiple factors, mechanisms, and linkages in the malignant cascade process. The pathological stimulation formed by cerebral ischemia or cerebral hemorrhage destroys the normal transmembrane ion gradient and neuronal homeostasis, causing many forms of cell death—including inflammation, oxidative stress, apoptosis, and autophagy. These pathological mechanisms cause significant damage in neurons, glial cells, and endothelial cells, all of which are core nodes in brain injury (72, 73). As one of the key regulatory mechanisms in stroke, ferroptosis can affect other biological processes or be triggered interactively in a positive-feedback loop so as to facilitate the survival of damaged neurons. Since ferroptosis itself reflects oxidative damage, it is not necessary to describe in detail the relationship between ferroptosis and oxidative stress. This paper will therefore emphasize the complex relationships between ferroptosis and inflammation, autophagy, and apoptosis (Figure 2).

### Ferroptosis and Inflammation in Stroke

Inflammation is known to be a protective stress measure generated by the body against tissue injury or infection, and it coordinates defense and tissue repair. In this process, damaged cells express a variety of inflammatory mediators such as cytokines, chemokines, and lipids and continuously recruit immune cells, trigger inflammatory positive feedback, and magnify local inflammatory responses (74). Inflammation is one of the critical steps in the onset and development of cerebrovascular diseases (75). As an important mechanism underlying brain injury, brain inflammation is recognized as an effective therapeutic target for some nervous system diseases such as ischemia or hemorrhage (76) and is a core factor affecting long-term prognosis after stroke. Although the pathogenic processes in primary injuries in ischemic and hemorrhagic stroke are different, the forms of immune activation are very similar. It was noted that after stroke, many factors, such as the production of ROS, necrotic cells, and damaged tissues, were combined, leading to the activation of damage-associated molecular patterns (DAMPs) (77, 78) and the release of a variety of inflammatory factors. These factors are detected by immune cells with corresponding pattern-recognition receptors, which then trigger inflammatory responses. In addition, the infiltration of blood components into the brain parenchyma also activates the immune response after intracerebral hemorrhage (79). However, in the late stage of stroke, the structure and function of the BBB are destroyed (80), promoting the infiltration and accumulation of peripheral leukocytes in the damaged site. Pro-inflammatory factors, ROS, and matrix metalloproteinases released by leukocytes then aggravate the damage to the BBB (81), forming a vicious cycle of effects.

The process of ferroptosis is also immunogenic and affects inflammation in many ways. Studies have confirmed that ferroptotic cells release inflammatory factors and DAMPs, forming a pro-inflammatory environment (82), and that ferroptotic activators—including erastin, sorafenib, RSL3, and FIN56—induce cancerous and non-cancerous cells to release high-mobility group box 1 (HMGB1) to amplify the inflammation (83). Ferroptosis can also effectively induce inflammation by releasing IL-33 and activating other as-yet identified pathways (13). In addition, ferroptosis accelerates the metabolism of arachidonic acid (AA); stimulates the synthesis of bioactive inflammatory mediators such as prostaglandin (PG), leukotriene (LT), epoxyeicosatrienoic acids (EETs), and hydroxyeicosatetraenoic acids (HETEs); and is critical to physiological processes and inflammatory responses (84). It is precisely because of the immune characteristics of ferroptosis that it can function in the progression of inflammatory diseases, including stroke.

Keuters et al. (85) demonstrated that ADA-409-052—as an effective ferroptotic inhibitor—can reduce lipid peroxidation, GSH depletion, and GPX4 inactivation, inhibit the pro-inflammatory activation of BV2 microglia, protect N2a neurons from inflammatory macrophage damage, down-regulate the expression of pro-inflammatory genes, shrink infarct volume, and mitigate edema in mouse models of stroke. Cui et al. (86) ascertained that overexpression of ACSL4 promoted ferroptosis and aggravated brain injury after ischemic stroke, while knockout of the ACSL4 gene inhibited the process of ferroptosis. This, in turn, inhibited the activation of microglia and the secretion of the inflammatory cytokines TNF, IL-6, and IL-1β; restricted the spread of inflammation; reduced the volume of cerebral infarction; and improved neurologic impairments. Similarly, Abdul et al. (87) revealed that DFO inhibited the activation of microglia in the prefrontal cortex and striatum, down-regulated inflammatory mediators such as TNF and CD16/32, promoted the expression of anti-inflammatory arginase 1, alleviated cerebrovascular degeneration and neuronal damage, reduced BBB permeability, and improved sensorimotor and cognitive impairment after stroke. Investigators have reported that an MCAO mouse model with mitochondrial ferritin (FtMt) deficiency showed characteristics typical of ferroptotic molecules, which promoted the inflammatory response induced by Imax R, while the overexpression of FtMt reversed these changes (88). In addition, Zhang et al. (89) observed that pyridoxal isonicotinoyl hydrazine (PIH) decreased iron accumulation, ROS production, and lipid peroxidation around hematomas; upregulated GPX4; inhibited pro-inflammatory polarization; down-regulated the expression of IL-1β and TNF; and improved the degree of neurologic impairment in mice after intracerebral hemorrhage. This suggests that there exists a close relationship between ferroptosis and neuroinflammation after intracerebral hemorrhage. Using an *in vivo* SAH model, liproxstatin-1 (Lip-1) not only promoted the activity of GPX4 and down-regulated ACSL4 but also inhibited the activation of microglia and attenuated the release of IL-6, IL-1β, and TNF to protect damaged nerve cells (90). Thus, inhibiting the onset of ferroptosis in nerve cells provides an effective means to reduce neuroinflammatory injury, improve outcome, and promote functional recovery after stroke.

### Ferroptosis and Autophagy in Stroke

Autophagy occurs via the regulation of autophagy-related proteins, with exfoliated endoplasmic reticulum and Golgi membrane combining with damaged organelles or abnormal proteins to form an autophagosome with a bilayer membrane structure that then fuses with lysosomes to form autophagic lysosomes. The lysosomal contents are then degraded by lysosomal enzymes in response to the metabolic needs of tissue cells or the renewal of some organelles (91). Studies have shown that autophagy is involved in the pathological underpinnings of stroke and that it can be observed in both hemorrhagic and ischemic brain tissues (92–94), thus playing a dual role. For example, the activation of autophagy during cerebral ischemia can promote neuronal survival (95, 96), and blocking autophagy with 3-methyladenine (3-MA) can also prevent ischemia-induced neuronal death and significantly reduce infarct volume (97, 98). Inhibition of autophagy aggravates brain injury after intracerebral hemorrhage (99), while activation of autophagy in the process of SAH significantly alleviates the disease (100). TLR4-induced autophagy, however, inhibits microglial activation and worsens inflammatory injury in intracerebral hemorrhage (101). Therefore, the role of autophagy in stroke remains controversial and requires additional clarification.

It is worth noting that ferroptosis interacts with autophagy by mediating autophagic activity within neurons. It was reported that the elevated expression of autophagy markers can be detected by injecting Fe^2+^ into the hippocampus of rats *in vivo* and exposing their embryonic cortical neurons to an external environment of ferrous iron *in vitro* (102). Ferroptotic activators such as erastin and RSL3 also induce the accumulation of ATG3, ATG5, ATG7, ATG13, and other autophagy genes in cells (103). In contradistinction, autophagy can also induce cellular ferroptosis; for example, research has uncovered augmented ferroptosis due to the up-regulation of autophagy genes (103) and has demonstrated that nuclear receptor co-activator 4 (NCOA4)-induced ferritin-mediated phagocytosis, RAB7A-dependent lipid phagocytosis, BECN1-induced systemic inhibition, STAT3-promoted lysosomal membrane permeability, and HSP90-related molecular chaperone-mediated autophagy may all lead to ferroptosis.

Experiments have verified that the interaction between ferroptosis and autophagy also occurs in stroke. Li et al. (104) found that the deletion of NCOA4 significantly eliminated ferritin-mediated phagocytosis induced by cerebral ischemia-reperfusion *in vivo* and *in vitro*, down-regulated ACSL4 and 15-LOX2, promoted the expression of GPX4 activity to inhibit ferroptosis, and reduced ischemic brain injury. Rong et al. (105) showed that ubiquitin-specific protease 11 (USP11) was up-regulated in mice with spinal cord ischemia-reperfusion injury (SCIRI) and autophagic activation and flux were increased and that the induction of additional autophagosomal formation led to ferroptosis by stabilizing Beclin1. Conversely, the inhibition of autophagy attenuated USP11-mediated ferroptosis, which was beneficial to functional recovery in SCIRI. Fang et al. (106) observed that compound 9a, a novel ferroptosis inhibitor, directly bound to the recombinant protein NCOA4^383−522^ and effectively blocked the interaction between NCOA4^383−522^ and FTH1, inhibited ferroptosis, and significantly improved cerebral ischemia-reperfusion injury. In addition, 17β-estradiol-3-benzoate attenuated the brain damage caused by iron overload by inhibiting autophagy after intracerebral hemorrhagic stroke (107). When ferrous citrate (FC) was injected into the brain tissue of male rats with intracerebral hemorrhage, the striatum showed a higher degree of autophagy and more severe brain injury, while ATG7 knockout reduced the autophagic injury induced by FC (108). Baicalin treatment down-regulated the expression of Beclin1 protein and microtubule-associated protein 1 light chain 3-II (LC3-II) and down-regulated the levels of Fe2+ and MDA in the brain of SAH rats; this suggested that baicalin exerted a brain-protective effect by inhibiting autophagy-dependent ferroptosis in SAH (109). Liang et al. (110) revealed that ferroptosis induced by SAH was autophagy-dependent. Knockout of the autophagy-related gene ATG5 inhibited autophagy, decreased the levels of intracellular iron and lipid peroxidation, augmented the levels of anti-ferroptosis protein, and significantly improved the prognostic index with SAH. The above data show that autophagy and ferroptosis exhibit an exquisite crosstalk and form a closed loop of brain injury in the process of stroke. Blocking one of these mechanisms can therefore enhance the survival outcome of nerve cells, and this is expected to develop into a novel treatment strategy for stroke.

### Ferroptosis and Apoptosis in Stroke

Apoptosis, as one of the classical forms of programmed cell death, is an important component of the pathological cascade in stroke (111); and it can be triggered by endogenous and exogenous pathways. The exogenous pathway begins when the extracellular death ligand activates the death receptor (DR), thereby activating caspase-8 and cleaving downstream caspase. With respect to the endogenous pathway, mitochondrial Ca^2+^ overload causes the cell membrane potential to collapse. Under the combined action of porogenic protein, the mitochondrial membrane permeability transition pore (MPTP) opens and releases cytochrome c (Cytc), apoptosis-inducing factor (AIF), and apoptotic-protease activating factor-1 (APAF-1) to form apoptosomes; this activates caspase-9, and AIF translocation to the nucleus causes DNA breakage and apoptosis in a caspase-independent manner. During this process, endogenous and exogenous pathways are often intertwined, leading to the activation of caspase-3 and apoptosis.

Ferroptosis is in fact closely related to the process of apoptosis. Cell experiments *in vitro* substantiated that GSH-depleted cells could not activate caspase normally (5), and Dixon posited that alterations in the content of biochemical molecules related to iron-induced cell death would inhibit the onset of the apoptotic process (112). In contradistinction, Park et al. (24) revealed the increased cleavage of caspase-3 due to ferroptosis induced by ferric ammonium citrate (FAC), while the use of deferric ammonium inhibited apoptotic damage. This complex relationship between ferroptosis and apoptosis also exists even after stroke.

Chen et al. (113) noted that overexpression of ferritin significantly reduced caspase-9 cleavage, inhibited hippocampal neuronal apoptosis, and alleviated ischemia-reperfusion injury in the brain of rats with MCAO by down-regulating the key regulatory factors of ferroptosis, p 53, and SLC7A11. Yigitkanli et al. (67) found that 12/15-LOX, as the core catalytic enzyme in lipid peroxidation in ferroptosis, induced apoptosis in the cortex and hippocampus of mice with transient global cerebral ischemia in both caspase-independent and caspase-dependent manners, while the lipoxygenase inhibitor LB-1 was used to reduce the number of neurons undergoing apoptosis. In addition, the results of Bao et al. (114) revealed an inhibition of FPN expression, an aggravation of local iron deposition, and an increase in neuronal apoptotic rate in a model of intracerebral hemorrhage, while overexpression of FPN reversed these effects, significantly reducing hematoma volume and improving neurologic function. Although it is not difficult to reveal the complex relationship between ferroptosis and apoptosis in stroke, the specific underlying mechanism(s) remains unclear, and there are few extant studies on their relationship. This, therefore, poses a new challenge to theoretical and clinical research endeavors dealing with stroke.

## Protective Effect of Inhibiting Ferroptosis in Stroke

Several studies have confirmed that ferroptosis is involved in many nervous system diseases such as Parkinson disease (PD) (15), Alzheimer disease (AD) (16), Huntington disease (HD) (17), ALS (115), traumatic brain injury (116), and periventricular leukomalacia (117). Ferroptosis has thus become one of the most important forms of cell death in nervous system diseases, and stroke is no exception.

The sesamin (SES)-treated MCAO mouse model was shown to inhibit ferroptosis and significantly reduce infarct volume by increasing the level of GSH and activity of superoxide dismutase (SOD) and by attenuating the expression of cyclooxygenase 2 (COX2), ROS, and lipid peroxide (118). Pretreatment of PC12 cells with low-dose bovine serum albumin (BSA) under OGD conditions enhanced the level of GSH and activity of SOD and thereby reduced the cytotoxicity induced by OGD (119). Phenothiazine derivatives can significantly inhibit the accumulation of ROS and malondialdehyde (MDA) induced by erastin *in vivo* and *in vitro*, increase the content of GSH, inhibit lipid peroxidation, and reduce ischemic brain injury (120). Frequency conversion in electroacupuncture intervention significantly decreased the content of total iron in ischemic brain tissues; up-regulated GSH, GPX4, and FTH1; down-regulated TF and TFR1; relieved the levels of oxidative stress; inhibited neuronal ferroptosis; improved motor dysfunction; and reduced the degree of cerebral infarction (121). Carthamin yellow (CY) protects the brain by reducing the accumulation of iron and ROS; down-regulating ACSL4 and TFR1; increasing the expression of GSH, GPX4, and FT in the brain; and inhibiting ferroptosis (122). Similarly, the inhibition of ferroptosis can also control the development of hemorrhagic stroke. Li et al. (123) revealed for the first time that ferroptosis was involved in collagenase-induced intracerebral hemorrhage in mice, and found that the ferroptosis inhibitor ferrostatin-1 (Fer-1) reduced iron deposition *in vivo* and *in vitro*; down-regulated ROS, prostaglandin-peroxide synthase (PTGS2), and COX2; prevented hemoglobin-induced neuronal death; and lessened neurologic and memory impairment and brain atrophy caused by intracerebral hemorrhage, thus revealing a long-term brain-protective effect (124). Fer-1 treatment of the SAH rat model up-regulated FPN, decreased iron content, improved lipid peroxidation, alleviated brain edema, and promoted neuronal survival (125). Baicalin can also inhibit ferroptosis induced by hemin, erastin, and RSL3; enhance cellular viability; and reduce hemorrhagic brain injury (126). Other studies have shown that the use of ferroptosis inhibitors curtailed neuronal mortality in hemorrhagic stroke by over 80% (127). These data show that ferroptosis mediates stroke injury, while the inhibition of ferroptosis reduces damage, and the fact that ferroptosis primarily occurs in neurons suggests that it constitutes an important potential target for stroke intervention.

## Summary and Future Directions

In summary, ferroptosis functions as one of the key pathological mechanisms subserving stroke and significantly affects the onset and development of the disease through abnormal changes in the metabolism of many related biochemical molecules; and the inhibition of ferroptosis may be significant in brain protection after stroke. As a novel form of cell death, ferroptosis provides a diverse view regarding our understanding of the mechanisms underlying brain injury after stroke. In addition, ferroptosis also participates in reciprocal crosstalk with the pathological processes inherent to inflammation, autophagy, and apoptosis involved in stroke. These processes collectively determine the outcome of damaged nerve cells, indicating that combined treatment entailing multiple mechanisms may block the pathological cascade and crosstalk in stroke. We posit that this creates a vicious cycle, achieving a curative effect of 1 + 1 > 2 as an authentic means toward resolving this disease. However, most of the current research data on ferroptosis in stroke come from animal experiments, and there is thus a lack of evidence for its clinical application. It remains unclear as to whether these compounds developed for ferroptosis will be effective, or whether they will produce side-effects in patients. Although the inhibition of the ferroptotic process is expected to become a new target in the treatment of stroke, there is still a paucity of sufficient direct data to support its correlation with clinical treatment of the disease. In addition, with the gradual emergence of a relationship between ferroptosis and other pathological processes, the fundamental mechanism of action of the former warrants further study. Therefore, based on the findings of this study and other previous studies, we will, in the future, carry out in-depth basic, clinical research and attempt to reveal the optimal treatment strategy so as to effectively regulate ferroptosis and to apply it to the treatment of stroke.

## Author Contributions

X-LF: collected, sorted out the literature, completed the writing of the manuscript, and the drawing of pictures. J-HW and X-LL: provided financial support for this study. X-ZD and J-HW: reviewed and revised the manuscript. S-YD: helped and supported X-LF's work. All authors contributed to the article and approved the submitted version.

## Funding

This review was supported by the National Natural Science Foundation of China (81960896) and Innovation Fund Project of Ministerial Universities in Gansu Province (2021A-210).

## Conflict of Interest

The authors declare that the research was conducted in the absence of any commercial or financial relationships that could be construed as a potential conflict of interest.

## Publisher's Note

All claims expressed in this article are solely those of the authors and do not necessarily represent those of their affiliated organizations, or those of the publisher, the editors and the reviewers. Any product that may be evaluated in this article, or claim that may be made by its manufacturer, is not guaranteed or endorsed by the publisher.
